# Effects of Multipolar Radiofrequency and Pulsed Electromagnetic Field Treatment for Face and Neck Rejuvenation

**DOI:** 10.1155/2017/4146391

**Published:** 2017-03-08

**Authors:** Thais Cristina Ferraz de Oliveira, Sheyla de Fatima Soares Rocha, Daniel Gontijo Ramos, Camila Gontijo Ramos, Michelle Vanessa dos Anjos Carvalho, Mariana Gontijo Ramos

**Affiliations:** ^1^Faculty of Human, Social and Health Sciences, Universidade Fumec, 30310-190 Belo Horizonte, MG, Brazil; ^2^Department of Dermatology, Santa Casa de Belo Horizonte, 30150-221 Belo Horizonte, MG, Brazil; ^3^Dermatology Clinic, 30110-921 Belo Horizonte, MG, Brazil; ^4^Faculty of Business Sciences, Universidade Fumec, 30310-190 Belo Horizonte, MG, Brazil

## Abstract

Skin aging is a gradual process that leads to wrinkle formation, laxity, and overall changes in skin appearance. In recent years, the demands to noninvasive treatments for facial rejuvenation increased, along with a variety of technologies and devices, such as radiofrequency. The present study aimed to evaluate the clinical effects of a multipolar radiofrequency and pulsed electromagnetic field treatment for face and neck rejuvenation. Eleven patients with mild to moderate grades of photoaging underwent eight radiofrequency and pulsed electromagnetic field treatment sessions, once a week. Clinical photographs were taken before and a week after the end of the treatment, and improvement of facial skin parameters was evaluated by two different investigators. Significant improvement in skin laxity was observed in all eleven patients (100%). Improvement in facial contour was noted in 73% and 100% of patients when analyzed by investigators A and B, respectively. The score for overall improvement in skin condition was 3 ± 0.78 for investigator A and 3.6 ± 0.67 for investigator B. All patients were satisfied with the procedure and noted significant improvement in the skin. The combined multipolar radiofrequency and pulsed electromagnetic field device is effective and safe for treatment of aged skin in Brazilian patients.

## 1. Introduction

The aging population is continuously increasing, and hence many people are seeking technologies and treatments to maintain skin health and a lasting youthful appearance. Treatments for many aesthetics aspects of the aging skin have, for many years, been different forms of surgery. However, busy lifestyle and technological development are constantly increasing the demand for nonsurgical skin rejuvenation procedures, with minimal risk and side effects and with rapid recovery time [1, 2].

Radiofrequency (RF) devices remain a dominant technology in the noninvasive management of skin aging, as it is a safe and effective treatment for a broad range of skin conditions. It can induce wrinkle reduction, cellulite improvement, laxity and body, and skin contouring improvement [3]. When radiofrequency is applied by an alternating current, an electric field is generated, which achieves skin tissues, generating thermal energy. The heat is not diminished by tissue diffraction or absorption by epidermal melanin and is then appropriate for treatment of all skin types [4].

Based on their number of electrodes, noninvasive RF devices can be categorized as monopolar, bipolar, tripolar, multipolar, and multigenerator. They can also combine different energy modalities in the same device, such as RF integrated with vacuum systems, infrared lights, lasers, and pulsed electromagnetic fields (PEMFs) [5, 6]. The radiofrequency heat seems to have different biological effects. The rise in skin temperature is able to cause immediate and temporary collagen shrinkage and increase the synthesis of collagen and elastin fibers by dermal fibroblasts. Thermal stimulation also induces augmented rate of lipase degradation of triglycerides to glycerol and free fatty acids [7].

Pulsed electromagnetic fields (PEMFs) are induced by short pulses of electrical current that penetrates into the skin and results in the stimulation of molecular and cellular activities. It has been used in medicine for bone growth, wound healing, cardiovascular disease, and other conditions [7]. Pulsed electromagnetic fields increase collagen fiber production by dermal fibroblasts and stimulate angiogenesis, leading to wound-healing effects [8].

A noninvasive device combines multipolar RF and PEMFs and is referred as (MP)^2^, which stands for “Multipolar Magnetic Pulse.” The device was introduced for the nonablative treatment of skin laxity and cellulite. The present study aimed to evaluate the clinical effects, including efficacy and adverse effects, of a combined multipolar radiofrequency and pulsed electromagnetic field treatment for face and neck rejuvenation.

## 2. Materials and Methods

Eleven subjects, one male and ten females, with mild to moderate grades of photoaging, meeting inclusion and exclusion criteria and providing signed informed consent, participated in this study. The study was reviewed and approved by the local ethics committee of the Universidade Fumec, Belo Horizonte, Brazil (protocol number 1.363.434).

Multipolar radiofrequency and pulsed electromagnetic field energy generating system (Venus Freeze, Venus Concept, Ontario, Canada) was used for treatment of face and neck. Patients were treated once a week for 8 weeks. The treatment area was cleaned with alcohol and dried, and pure glycerin was applied over the entire face and neck. The treatment parameters were determined depending on the area of treatment and patient skin type. During the treatment, the energy was adjusted as tolerated by the patient, with the goal to reach at least 40–42°C, and the temperature was kept constant during the treatment period, which was 20 to 30 minutes for each session. The applicator was gently and continuously moved on the skin surface and the skin temperature was measured using an infrared thermometer. Patients were told not to wash the face for 2 hours after treatment.

For treatment outcome evaluation, photographs were taken before and one week after the last treatment session. The photographs were taken with the patient wearing no make-up, sat on a chair and staring at the same point, in a controlled position. The pretreatment and posttreatment photographs were evaluated and graded by two separate investigators. Investigator A was a dermatologist, blinded to the study, and investigator B was a physical therapist that evaluated the photographs, but also the patients by direct skin observation during and after the treatment sessions. The investigators evaluated the improvement of the photoaged skin based on laxity, contour, wrinkles, and skin texture. The grade of improvement for each parameter was divided into three categories: worsened, no change, and improved. They also graded the overall clinical skin improvement using a previously described [9] Global Aesthetic Improvement Scale (GAIS) that was dived in five categories, from 1 to 5. One (1) = worse, two (2) = no change, three (3) = improved, four (4) = much improved, and five (5) very much improved. Treatment safety was evaluated by assessing side effects including erythema, edema, burn, and stinging sensation.

## 3. Results

Eleven patients were treated with RF and PEMF for eight weeks, and laxity, facial contour, wrinkles, skin texture, and overall skin improvement were assessed by two investigators using before and after photographs and the GAIS scale. One patient (9%) was male and ten (81%) were female, with mean age of 53.1 ± 7.4 years (range from 42 to 65 years). Five and six patients had Fitzpatrick skin types II and III, respectively (Table 1).

**Figure 1:**
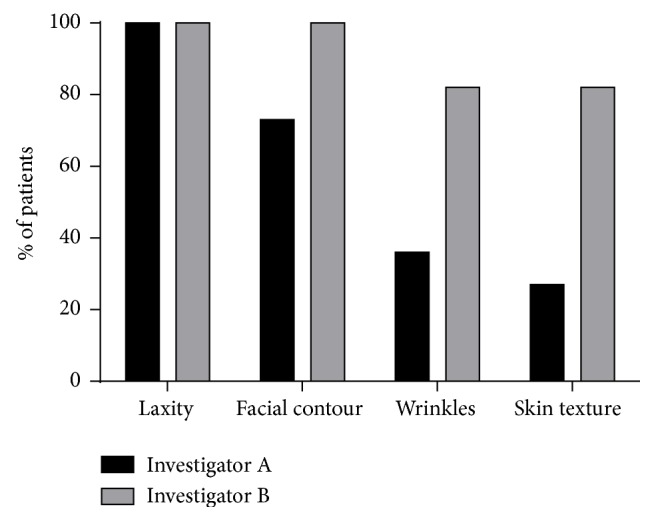
Professional evaluation of clinical improvement for different skin parameters. Data are expressed as percentage of patients showing improvement.

No patients showed a worsened appearance after the treatment. Both investigators agreed that all the eleven patients (100%) showed improvement of their skin laxity. Eight patients (73%) showed improvement of their facial contour as assessed by investigator A and eleven patients according to (100%) by investigator B. Investigator A also observed improvement of wrinkles in four patients (36%) and of skin texture in three patients (27%). Regarding investigator B, improvement was noted in nine patients (82%) for both wrinkles and skin texture (Figure 1).

The investigators also evaluated overall clinical skin improvement using the GAIS scale. Investigator A observed that three patients (27%) had improved results and eight patients (73%) had much improved results in overall skin condition after treatment. Investigator B analysis showed that for 10 patients (91%) a much improved skin condition was observed, and for one (9%) patient a very much improvement was seen (Table 2).

**Figure 2:**
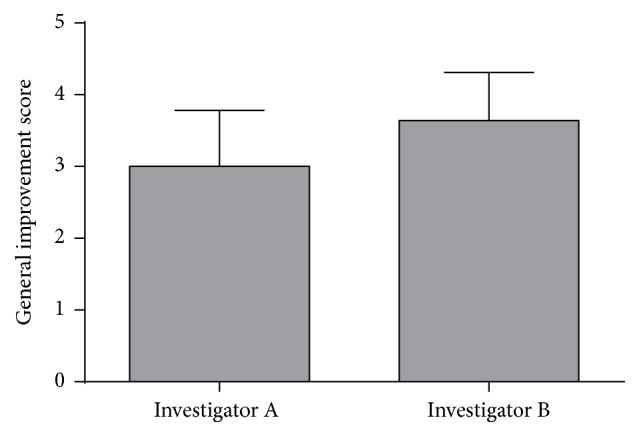
Professional evaluation of overall clinical skin condition improvement. Data are expressed as mean ± standard deviation of a 0 to 4 Global Aesthetic Improvement Scale (GAIS).

**Figure 3:**
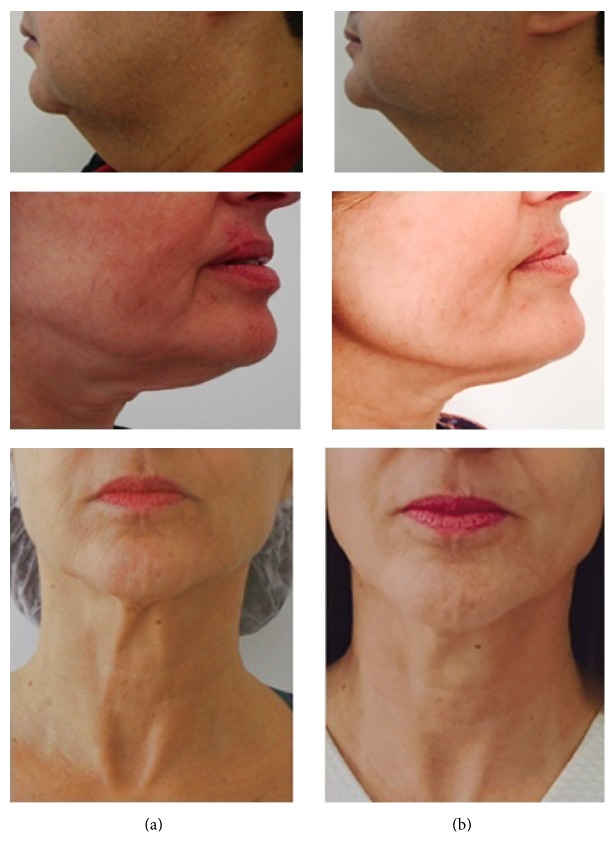
Improvements in skin condition. Photographs of selected patients before (a) and after eight sessions (b) of treatment with RF and PEMFs.

The mean score for clinical skin improvement was 3 ± 0.78 for investigator A and 3.6 ± 0.67 when assessed by investigator B (Figure 2).  Figure 3 shows representative photographs of clinical skin changes. A 55-year-old male patient showed change of neck contour after eight sessions. A 52-year-old and another female 55-year-old patient also showed improvement on face and neck contour and texture and substantial improvement of skin laxity (Figure 3).

Treatment was well tolerated by all patients and no significant pain or discomfort was reported. The main side effects observed were slight erythema and skin tightening that occurred immediately after treatment but resolved soon. No complications were observed during or after treatment. All patients reported that they were satisfied with the procedure and noticed visible improvement in their skin condition after the treatment.

## 4. Discussion

The results of this study suggest that the multipolar RF and PEMF device can provide satisfactory results for treating photoaged skin. This combined technology is different from the use of radiofrequency alone, as it delivers RF energy with the simultaneously addition of pulsed electromagnetic field. The synchronized treatment allows the delivery of more energy to the treated area, achieving higher temperature with minimal risk and pain, maintaining the epidermis intact, and leading to less side effects and shorter recovery periods, when compared to RF alone [1, 2].

The present study shows that all patients described the treatment as comfortable, with almost no pain and no undesirable side effects. They were also able to observe visible changes on their skin appearance and reported to be satisfied with the treatment outcomes.

Two investigators evaluated the improvement in skin parameters and overall skin condition before and after the eight sessions of treatment. No patients showed a worsened appearance after the treatment. Both investigators agreed that skin laxity was improved in all patients (100%). Facial contour was also improved in most patients (73% by investigator A and 100% by investigator B). Improvement in wrinkles and skin texture were less evident but could be noted in some patients. However, the results of the assessment of these two parameters were different between the two investigators. Investigator A that was blinded to the study and only evaluated before and after treatment photographs recorded poorer results when compared to investigator B that analyzed the photographs but also directly observed the patient's skin before, during, and after treatment.

In the present study, the overall changes in skin condition evaluated by the Global Aesthetic Improvement Scale indicated that most of the patients showed an improved or much improved skin condition. The mean score for improvement was higher when evaluated by investigator B when compared to investigator A. These findings are important to show that changes in some parameters, such as laxity and facial and neck contour, are easier to be measured by before and after photographs observation, but changes in other parameters such as wrinkles and skin texture are harder to be evaluated by photographs. It also demonstrates that the clinical evaluation is somewhat subjective and can differ between investigators. Besides that, it is possible to suggest that RF and PEMF are capable of promoting important changes in photoaged skin, improving its laxity and appearance.

Despite few clinical studies using the same RF and PEMF technology, some other groups had also observed significant improvement in skin appearance after treatment. Lee et al. (2014) treated 10 Korean female patients with RF and PEMF and showed a substantially improved skin texture (70%) and skin laxity (50%), similar to what we observed in our study. However, different from our study, they noted only slight improvement in facial contour (25%) [10]. These variations could be explained by differences in ethnicities and ages of the treated patients, as Asian skin tends to age differently, with delayed wrinkle formation, compared to Caucasian skin. Younger patients also have less changes in skin overall appearance than older ones, and so the changes are less evident and harder to be noticed. In another study, thirty-one subjects with facial wrinkles and rhytides were treated with a similar device, and a significant decrease in Fitzpatrick Wrinkle and Elastosis Scale (FWES) was demonstrated [11]. A study demonstrated that RF and PEMF can also be effective for body treatment, in areas such as abdomen, flanks, arms, and legs [12]. The authors showed a significant reduction in visibility and in width and length of striae (stretch marks) in female patients.

Application of radiofrequency (RF) current to the skin is supposed to be able to modulate its mechanical properties, inducing immediate and long-term effects and consequently leading to improvement in the skin condition [4]. Immediate effects are mediated by heat disruption of hydrogen bonds in the triple helix collagen structure, leading to partial protein denaturation [13]. RF can increase local blood flow, upregulating local adipose metabolism, and is capable of stimulating lipase-mediated degradation of triglycerides or even adipocytes apoptosis [4]. Delayed effects include thermal induced microinflammatory response in skin tissue, leading to neocollagenesis, which is the result of dermal remodeling to decompose damaged collagen by collagenase enzyme, and replace it with new collagen. Elastin and ground substance production is also stimulated [14, 15].

The results achieved with RF treatments depend on several factors, including patient individual characteristics, gender, age, degree of photoaging, and skin phototype [16]. Improvement of skin structure can also vary according to treated facial area, because different biological tissues have variable levels of impedance [4, 17].

Although RF and other treatments to improve skin condition are shown to be efficient, relapse is inevitable, since skin aging is a natural process, and additional treatment could be required, according to the patient's skin condition and demand, in order to keep the results [18].

Radiofrequency technologies continue to evolve, with many new devices available in the market. Fractional radiofrequency (electrode pin and microneedle) has recently been developed to further improve efficacy and safety of skin rejuvenation therapies [14, 19]. With so many diverse treatment options, it is important to understand the differences between the classes of RF devices and to try to achieve the best approach to specific needs, according to individual patients.

There were some limitations of this study. The sample size was small, and long-term follow-up was not assessed. The degree of skin improvement was measured by photographs observation, and objective measurements, using instruments, was not performed. However, it is a common clinical practice to evaluate results of dermatological procedures by photographs analysis, as skin measurement instruments are not always available.

## 5. Conclusion

The results of the present study demonstrated the clinical effects of the combined multipolar RF and PEMF device for skin rejuvenation. The use of RF and PEMF is safe and effective for treating aged skin. Treatment improved skin condition, decreasing skin laxity, attenuating wrinkles, and smoothing facial and neck contour, with no pain or undesirable side effects.

## Figures and Tables

**Table 1:** Demographic characteristics of the treated patients.

*Gender*	
Male (*n* = 1) Female (*n* = 10)	9% 81%
*Age (mean ± SD, range)*	53.1 ± 7.3 (42–65)
*Fitzpatrick phototype*	
II (*n* = 5) III (*n* = 6)	45% 55%

**Table 2:** Overall clinical skin improvement evaluation by two separate investigators.

Improvement	Investigator A *N* (%)	Investigator B *N* (%)
Worse	0 (0%)	0 (0%)
No change	0 (0%)	0 (0%)
Improved	3 (27%)	0 (0%)
Much improved	8 (73%)	10 (91%)
Very much improved	0 (0%)	1 (9%)

The Global Aesthetic Improvement Scale (GAIS) was used for skin improvement evaluation. Worse (0), no change (1), improved (2), much improved (3), and very much improved (4). *N* = number of patients.
